# Improved image reconstruction incorporating non-rigid motion correction for cardiac MRI using BLADE acquisition

**DOI:** 10.1186/1532-429X-11-S1-P227

**Published:** 2009-01-28

**Authors:** Peter Kellman, Magalie Viallon, Christophe Chefd'hotel, Alto Stemmer, Pierre Croisille

**Affiliations:** 1 NIH, Bethesda, MD USA; 2 University Hospital of Geneva, Geneva, Switzerland; 3 Siemens Corporate Research, Princeton, NJ USA; 4 Siemens Medical Solutions, Erlangen, Germany; 5 University Hospital of Cardiology Louis Pradel, Lyon, France

**Keywords:** Full Resolution, Dark Blood, Image Reconstruction Method, Respiratory Motion Artifact, Full Resolution Image

## Introduction

The application of the BLADE sequence for dark blood T2-weighted cardiac MRI has the potential for increasing the spatial resolution thereby improving the detailed depiction of morphology [1]. While the BLADE sequence using periodically rotating overlapping parallel lines (PROPELLER) [2] inherently provides a degree of motion tolerance, significant respiratory motion may result in image artifacts. Rigid body motion correction applied to PROPELLER MRI [2] is well suited to applications such brain imaging but is problematic in cardiac MR where the motion is not rigid. Non-rigid image registration has been successfully applied for motion corrected averaging in cardiac MR [3]. In the present study, non-rigid motion correction was applied to individual BLADE images prior to combination as a high resolution image, to migitate respiratory motion artifacts that arise despite the use of a navigator.

## Purpose

To develop and evaluate an improved image reconstruction method for cardiac MRI acquired using a navigated BLADE sequence.

## Methods

Dark blood prepared, navigated, ECG-gated T2-weighted cardiac MR imaging was performed using a BLADE sequence with TSE readout. Typical parameters for imaging using the Siemens Magnetom AVANTO 1.5 T scanner were: echo train length = 26, echospacing = 6 ms, TE = 78 ms; #BLADES = 17, readout resolution = 256, PE resolution = 44 per BLADE after rate 2 parallel imaging using 8 central reference lines, typical FOV 320 × 320 with 1.25 × 1.25 mm^2^ in-plane resolution with 6 mm slice thickness.

**Figure 1:**
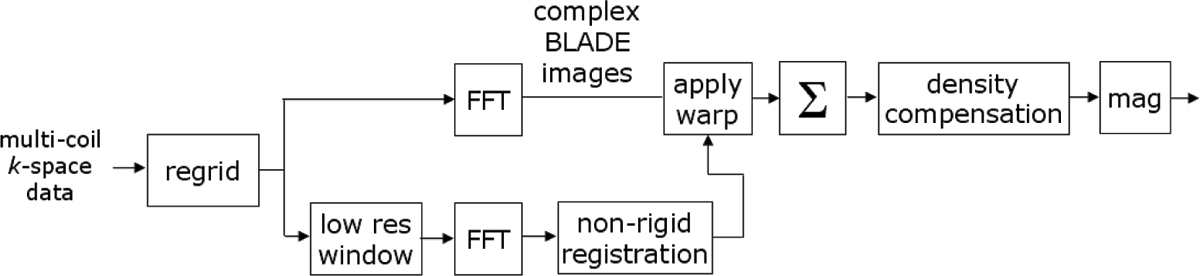
**Motion corrected BLADE image reconstruction diagram (simplified)**.

Imaging reconstruction (Figure 1) was performed off-line from raw data using MATLAB. Sub-pixel non-rigid image registration [4] was performed on the individual low resolution BLADE magnitude images (following parallel imaging reconstruction) and the resultant motion field was applied to the full resolution complex BLADE images prior to combining as a full resolution image (256 × 256). Density correction was then applied to the full resolution image.

## Results

**Figure 2:**
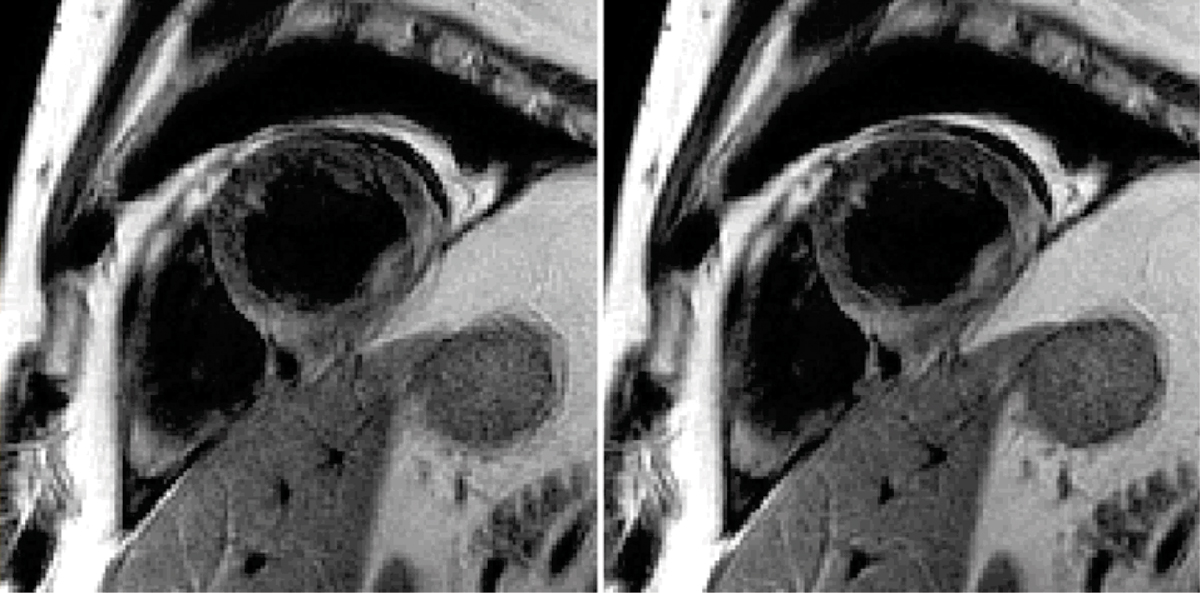
**Example of a dark-blood prepared TSE BLADE image showing conventional (left) and motion corrected (right) reconstruction for a case in which there are no respiratory image artifacts**.

**Figure 3:**
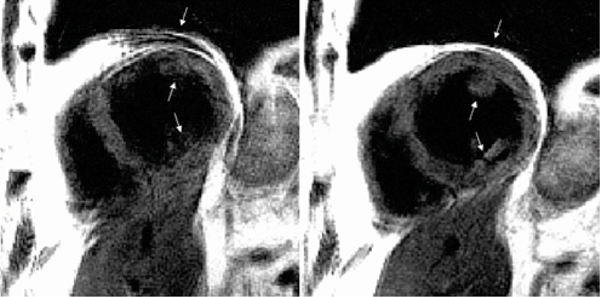
**Example of a dark-blood prepared TSE BLADE image showing conventional** ***(left)*** **and motion corrected** ***(right)*** **reconstruction for a case in which there are significant respiratory image artifacts in conventional reconstruction (indicated by arrows)**.

Motion corrected and conventional BLADE reconstructions were compared for a number of slices for N = 11 patient studies. These studies included cases for which conventional reconstructions were of good quality (Figure 2) as well as several cases which exhibited respiratory motion artifacts (Figure 3). The proposed method did as well or better in all cases with significant artifact reduction in several cases.

## Conclusion

The proposed approach for BLADE reconstruction using non-rigid motion correction significantly improves the image quality in cases with respiratory motion artifacts. The method is fully automatic not requiring any user interaction to define bounding regions of interest.

## References

[1] Viallon M (2008). Proceedings ISMRM.

[2] Pipe J (1999). Magn Reson Med.

[3] Ledesma MJ (2007). J Magn Reson Imaging.

[4] Chefd'hotel C (2002). Proc IEEE ISBI.

